# Impact of claw lesions on dairy cow energy-corrected milk yield: Differential and persistent effects up to 120 days post-treatment

**DOI:** 10.1016/j.vas.2026.100605

**Published:** 2026-02-21

**Authors:** Zdeněk Havlíček, Lucie Langová, Irena Vrtková, Petr Doležal, Petr Kouřil, Katarzyna Szwedziak

**Affiliations:** a Department of Morphology, Physiology and Animal Genetics, Mendel University in Brno, Faculty of AgriSciences, Zemědělská 1/1665, 613 00 Brno, Czech Republic; b Department of Animal Breeding, Animal Nutrition & Biochemistry, University of Veterinary Sciences Brno, Faculty of Veterinary Hygiene and Ecology, Palackého tř. 1946/1, 612 42 Brno, Czech Republic; c Department of Animal Nutrition and Forage Production, Mendel University in Brno, Faculty of AgriSciences, Zemědělská 1, Brno, Czech Republic; d Department of Food Technology, Mendel University in Brno, Faculty of AgriSciences, Zemědělská 1/1665, 613 00 Brno, Czech Republic; e Akademia Nauk Stosowanych Angelusa Silesiusa, ul. Zamkowa 4, 58-300 Wałbrzych, Poland

**Keywords:** Lameness, Claw lesions, Dairy cattle, Milk yield, Digital dermatitis, Toe ulcer, White line disease, Farm profitability, Animal welfare

## Abstract

Claw lesions can substantially reduce milk production in dairy cows, yet quantification by lesion type and disease phase is limited. We analysed 5,947 daily milk yield records from 818 cows to evaluate the impact of specific claw lesions on energy-corrected milk (ECM), considering lesion type and disease phases (pre-treatment, treatment, post-treatment). Mixed linear models revealed significant effects of the diagnosis × phase interaction (*P*<0.001) and lactation order (*P*<0.001). Healthy cows maintained a stable LS Mean ECM of 33.91±0.19 kg/day. The largest production loss occurred in interdigital phlegmon during treatment (reduction of 4.8 kg/day relative to healthy cows). Reductions were also observed in digital dermatitis (DD), white line disease (WL), sole ulcer (SU), toe ulcer (TU), and combination lesions, with persistent deficits for some claw horn lesions post-treatment (e.g., SU: *β*=–1.9, *P*=0.05). A significant pre-treatment elevation in ECM observed for white line disease (+2.4 kg/day) likely reflects the higher baseline production and increased susceptibility of these individuals to claw lesions, rather than a beneficial effect of the pre-clinical disease phase. These findings highlight the importance of early detection, effective treatment, and preventive claw trimming and claw care to maintain milk production, minimise economic losses, and ensure animal welfare.

Interpretive Summary: We evaluated how specific claw lesions influence milk production across disease phases using daily records. Milk output typically fell during treatment and partially recovered post-treatment. However, several claw-horn lesions exhibited more durable deficits, suggesting lingering effects beyond clinical resolution. The pattern indicates that some conditions cause a brief, acute production loss, whereas others impose longer-lasting penalties. These insights support earlier recognition, targeted therapy, and preventive management—claw trimming, flooring, cow flow, and comfort—to maintain yield, limit economic losses, and improve welfare.

## Highlights

• Specific hoof lesions cause diagnosis-specific milk yield depressions.
• Interdigital phlegmon causes the largest acute ECM loss (4.8 kg/day).
• Production deficits for sole ulcers persist up to 120 days post-treatment.
• High-yielding cows show greater vulnerability to claw horn lesions.
• Hoof diseases act as systemic challenges beyond local clinical signs.

## Graphical abstract

**Figure:**
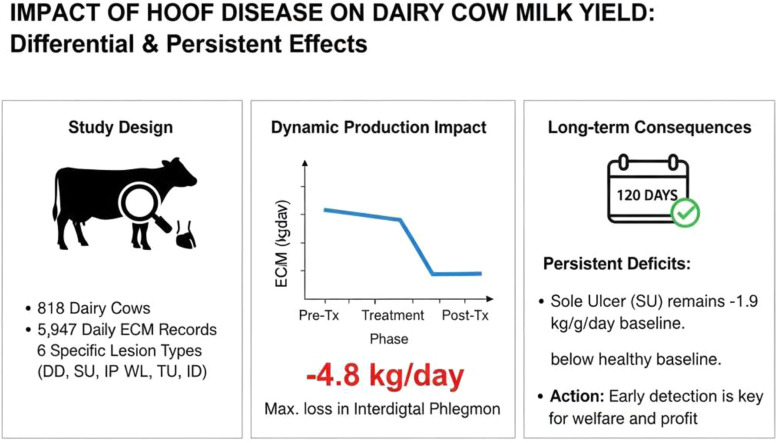

## Introduction

Globally, the prevalence of lameness in high-yielding dairy herds ranges from ∼20% (Solano et al., 2015) to more than 30%, and in some confined housing systems even above 50% (von Keyserlingk et al., 2012). This variability is strongly influenced by housing and management; for instance, herds in freestall barns with concrete flooring typically show higher prevalence compared to those on pasture or deep-bedded packs. In the European context, recent benchmarking of Austrian dairy herds revealed a mean lameness incidence risk (LSC ≥ 2) of 38.3%, with significant variation between the 'best-in-class' farms (16.2%) and those in the 90th percentile (61.3%), highlighting the substantial potential for management-led improvements (Kofler et al., 2022). Recent reviews indicate an overall prevalence of approximately 22–23% in modern dairy herds (Thomsen et al., 2023), underscoring the persistence of this challenge worldwide. Such high prevalence emphasises the dual imperative of maintaining milk production while safeguarding animal welfare. From an economic perspective, claw lesions are among the costliest production diseases, with total losses being highly variable, ranging from €43 to over €1,040 per case depending on the etiology and management efficiency (Robcis et al., 2023a). In terms of overall impact, claw disorders represent the third largest economic loss in dairy units after mastitis and reproductive disorders (Ózsvári, 2017). These losses arise not only from reduced milk yield but also from extended calving intervals, increased culling risk, veterinary interventions, and decreased longevity, with every additional week a cow spends in a lame state (LSC ≥ 3) costing an average of €12.1 (Robcis et al., 2023a).

Lameness is not caused by a single disease but rather a clinical manifestation of various infectious (e.g., digital dermatitis) and non-infectious claw disorders (e.g., toe ulcers). The etiology of these conditions is multifactorial, with major contributing factors including genetics (Ring et al., 2018), management and housing conditions (Ózsvári, 2017; Robcis et al., 2023a), nutrition (Ózsvári, 2017), and oxidative stress (Abuelo et al., 2013). Each condition has distinct pathophysiology, degrees of severity and pain, leading to heterogeneous impacts on performance. Notably, so-called 'alarm lesions'—painful conditions such as ulcers, white line abscesses, and acute digital dermatitis—have been reported with a mean incidence risk of 30.1% in European herds, necessitating rapid clinical intervention (Kofler et al., 2022). Nevertheless, most previous studies have treated lameness as a generalised condition (Bicalho et al., 2008; Thomsen et al., 2023; Džermeikaitė et al., 2025). This generalisation constrains precise quantification of economic losses and hinders the development of targeted preventive strategies. Recent work emphasises the importance of distinguishing lesion types and disease phases to accurately quantify the magnitude of production losses (Green et al., 2010; Randall et al., 2015). Furthermore, bioeconomic modelling suggests that farm profitability can be optimized by strategic time allocation, specifically by prioritizing preventive measures such as footbath applications—ideally 17.8 to 22.3 hours per month for a 100-cow herd—over simple visual detection (Robcis et al., 2023a).

Lameness also affects behaviour, rumination, milk quality, and systemic metabolism (Necula et al., 2024), and even the environmental footprint of dairy production (Chen et al., 2016). Notably, these behavioural and physiological changes often precede clinical signs, highlighting the importance of early detection. Advanced precision technologies, including automated activity monitors and machine learning algorithms, are increasingly being proposed as tools for early identification of subclinical cases (Neupane et al., 2024). Given the significant economic impact of both lameness and mastitis as major production diseases (Ózsvári, 2017; Robcis et al., 2023a), and their potential systemic overlap, it is crucial to control for udder health status, typically assessed via somatic cell count (SCC), to accurately isolate the impact of claw lesions on milk yield. Although it is well established that lameness reduces milk yield, the detailed quantification of losses by specific diagnosis and disease phase (pre-treatment, treatment, and post-treatment) remains lacking. Such analyses are essential for optimising management and minimising economic losses. Therefore, this study aims to quantify milk yield losses—specifically energy-corrected milk (ECM)—in relation to specific claw lesions and disease phases, using each cow’s pre-disease baseline as a reference. This approach enables the assessment of both acute and persistent effects, providing novel insights into the long-term consequences of lameness on productivity and herd economics.

## Material and methods

### Data collection and study population

This retrospective study was conducted on a single large-scale commercial dairy farm in the Czech Republic. The herd was housed in a loose-stall (freestall) system with solid concrete flooring in the alleys and rubber mats in the lying stalls. To maintain claw health, a professional claw trimmer performed routine functional trimming twice per lactation (typically at dry-off and mid-lactation). Furthermore, clinical cases were identified and treated daily by trained farm personnel under the long-term supervision of a senior claw trimmer. A total of 5,947 daily milk-yield records from 818 dairy cows monitored between January 2017 and December 2024 were analysed. Detailed daily milk yield data were available for all 818 cows throughout the study period. Data were obtained from the farm’s breeding and herd management software, Farmsoft (Agro-soft Tábor, s.r.o., Tábor, Czech Republic), and from laboratory milk analyses.

For each cow, records included daily milk yield (kg), milk composition (percentages of fat, protein, and lactose), the logarithm of somatic cell count (LogSCC), urea level, and detailed information on claw lesions diagnoses (the term is used consistently throughout the study), including dates of diagnosis and treatment.

### Claw health monitoring and diagnosis

The claw lesions diagnoses were recorded electronically by professional claw trimmers and trained farm personnel using the Farmsoft documentation module. During the extended eight-year observation period, claw health was assessed during regular claw trimming sessions (conducted at least twice per year for all cows) and during individual clinical interventions for cows showing signs of lameness.

To ensure diagnostic consistency, the farm followed a standardized protocol based on the ICAR Claw Health Atlas. While a formal interrater reliability test was not conducted due to the retrospective nature of the study, the quality and consistency of the records were maintained through long-term supervision by the same senior professional claw trimmer throughout the entire observation period.

### Definition and categorization of variables

For analysis, diagnoses were grouped into eight categories: toe ulcer (TU), sole ulcer (SU), white line disease (WL), interdigital phlegmon (IP), interdigital dermatitis (ID), digital dermatitis (DD), combination of diseases (C), and Healthy (H) as a control. The ‘Combination of diseases (C)’ category was established to account for cows presenting with two or more distinct claw lesions simultaneously on one or more limbs during a single examination. This category primarily included concurrent infectious and non-infectious lesions, most frequently the presence of digital dermatitis (DD) alongside a claw horn lesion such as a sole ulcer (SU) or white line disease (WL). It also encompassed cases with multiple claw horn lesions, such as concurrent SU and TU on the same or different feet. By grouping these multi-lesion cases into a separate category, we ensured the independence of observations in the model and prevented the double-counting of individual cows, which would otherwise violate the assumptions of the mixed-effects analysis.

As part of the data collection, performance records were also extracted for the pre-treatment period, defined as the 14 days preceding the diagnosis for each affected cow. Values from this period served as individual baselines, allowing each cow’s production at the time of disease to be compared with her own baseline performance and thereby minimising the influence of individual genetic and management factors. However, it is acknowledged that subclinical claw disease can affect milk yield well before clinical diagnosis. Research by Green et al. (2002, 2010) indicated that milk yield reductions can be detected as early as four months before a clinical case is formally diagnosed. Therefore, our 14-day baseline values may already reflect some degree of production loss, potentially leading to a conservative estimation of the total cumulative loss. Nevertheless, this 14-day window was selected to capture the acute changes associated with the transition to the clinical phase and to ensure high data consistency across the study period.

The following variables were included in the main statistical model:

Dependent variable: Energy-Corrected Milk (ECM) yield (kg/day).

Independent variables (fixed effects):

Diagnosis: Categorical variable with nine levels (H, TU, SU, WL, IP, ID, DD, and C). Cases where a cow presented with two or more concurrent claw lesions during a single examination were consistently assigned to the 'Combination of diseases (C)' category. This approach ensured the independence of observations in the model and prevented the double-counting of individual cows.

Disease phase: Categorical variable defined as ‘pre-treatment’ (baseline, days -14 to -1), ‘treatment’ (days 0 to 21), and ‘post-treatment’ (days 22 to 120 after diagnosis). The post-treatment phase was defined to capture the long-term recovery trajectory of cows, particularly those with claw-horn lesions. Since horn tissue regrowth is a slow physiological process (Randall et al., 2015), a shorter window might have failed to detect the full duration of production deficits. We acknowledge that recovery may not be strictly linear within this 100-day window, but this categorization allows for a robust assessment of the overall economic impact on the remainder of the lactation.

Covariates (fixed effects):

Lactation order: Categorical variable (1st, 2nd, 3rd, 4th, and 5+ lactations).

Lactation phase (Days in Milk, DIM): Continuous variable to account for the physiological lactation curve.

Udder health (LogSCC): Log-transformed somatic cell count (Guzzo et al., 2018).

Nutritional indicator: Urea content in milk (continuous variable).

### Statistical analysis

The primary dependent variable for quantifying production losses was energy-corrected milk (ECM) yield (kg/day). ECM was calculated from daily milk yield and its composition (fat and protein percentages) using the standard formula proposed by Tyrrell and Reid (1965):

$$ECM=\frac{milk\;yield\;x\;\left(0.0929\;x\;fat\;\left(\%\right)+0.0563\;x\;protein\;\left(\%\right)+0.2520\right)}{3.14}$$

Raw milk yield (kg/day) and fat-corrected milk (FCM) were analysed as secondary dependent variables.

We analysed the data using a mixed linear model (PROC MIXED; SAS 9.4, SAS Institute Inc., Cary, NC, USA). The model included Diagnosis, Disease phase, and their interaction (Diagnosis × Phase) as fixed effects. The reference category for calculating least-squares means (LS means) was set to “Healthy”. To account for repeated measurements within individual cows over time, we applied a compound symmetry (CS) correlation structure for the residuals. The CS structure was selected because it provided the best fit for our data based on the Akaike Information Criterion (AIC) compared to other structures, such as first-order autoregressive (AR(1)). This structure assumes a constant correlation between any pair of observations from the same cow, regardless of the time interval between them.

Type III tests of fixed effects were used to assess statistical significance, with a threshold of *P*<0.05. Pairwise comparisons of LS means were performed using the Tukey–Kramer test, which adjusts for multiple comparisons and is suitable for evaluating all possible pairwise differences after a significant interaction effect. This approach ensures that the resulting *P*-values are reliable despite the large number of comparisons. All analyses were conducted strictly using available production and health records; additional covariates such as body condition score, energy balance, or heat stress were not included because they were not measured in this study.

## Results

The estimated fixed effects are summarised in Table 1, Table 2, Table 3, Table 4. Table 1 reports the Diagnosis × Disease phase interaction; Table 2 presents effects of lactation order (parity); Table 3 summarises effects of lactation phase (days in milk, DIM); and Table 4 shows effects of continuous covariates, including milk urea content (MUC) and log-transformed somatic cell count (LogSCC). Statistically significant contrasts (*P*<0.05) are highlighted, indicating that both disease status and lactation characteristics contribute to variation in energy-corrected milk (ECM). Overall, the results are consistent with a transient treatment-phase depression in ECM, alongside parity- and DIM-related differences across the herd.

**Table 1:** Estimated fixed effects for the Diagnosis × Disease phase interaction on energy-corrected milk yield (ECM, kg/day), with Healthy as the reference diagnosis and pre-treatment as the reference phase.

Diagnosis	Phase	*β*	*SE*	*DF*	*t value*	*P*	*Lower 95% CI*	*Upper 95% CI*
**Digital dermatitis**	Pre-treatment	0.5	0.38	5362	1.35	0.18	-0.23	1.27
	**Treatment**	**-0.7**	**0.30**	**4211**	**-2.26**	**0.02**	**-1.28**	**-0.09**
	**Post-treatment**	**1.3**	**0.34**	**3816**	**3.91**	**<0.001**	**0.67**	**2.01**
**Interdigital dermatitis**	Pre-treatment	-0.4	0.77	5849	-0.53	0.60	-1.90	1.10
	**Treatment**	**-0.9**	**0.55**	**5242**	**-1.70**	**0.09**	**-2.02**	**0.15**
	Post-treatment	0.5	0.63	5166	0.74	0.46	-0.77	1.69
**Interdigital phlegmon**	Pre-treatment	-1.8	1.73	4709	-1.06	0.29	-5.22	1.55
	**Treatment**	**-4.8**	**1.93**	**5375**	**-2.47**	**0.01**	**-8.56**	**-0.98**
	Post-treatment	-0.7	1.70	2054	-0.41	0.68	-4.03	2.63
**White line disease**	**Pre-treatment**	**2.4**	**0.92**	**5918**	**2.64**	**0.01**	**0.63**	**4.25**
	**Treatment**	**-1.6**	**0.64**	**3879**	**-2.54**	**0.01**	**-2.89**	**-0.37**
	**Post-treatment**	**-1.4**	**0.73**	**4906**	**-1.94**	**0.05**	**-2.83**	**0.01**
**Sole ulcer**	Pre-treatment	0.2	1.01	5907	0.22	0.83	-1.76	2.21
	**Treatment**	**-2.4**	**0.69**	**5318**	**-3.43**	**<0.001**	**-3.72**	**-1.02**
	**Post-treatment**	**-1.9**	**0.97**	**4809**	**-1.96**	**0.05**	**-3.80**	**0.00**
**Toe ulcer**	Pre-treatment	1.1	0.83	5916	1.35	0.18	-0.51	2.76
	**Treatment**	**-2.3**	**0.58**	**4586**	**-4.05**	**<0.001**	**-3.48**	**-1.21**
	Post-treatment	-0.2	0.66	4290	-0.24	0.81	-1.46	1.14
**Combination**	Pre-treatment	0.8	1.14	5861	0.70	0.48	-1.43	3.04
	**Treatment**	**-1.6**	**0.74**	**4315**	**-2.11**	**0.03**	**-2.99**	**-0.11**
	Post-treatment	-0.4	0.89	5205	-0.44	0.66	-2.14	1.35
Intercept	**Healthy**	**40.4**	**0.75**	**4039**	**54.23**	**<0.001**	**38.98**	**41.91**

Note: Positive β indicates higher ECM relative to the reference; negative β indicates lower ECM. Statistically significant effects (P<0.05) are highlighted in bold. β = estimate; SE = standard error; DF = degrees of freedom; CI = confidence interval.

**Table 2:** Estimated fixed effects for lactation order on energy-corrected milk yield (ECM, kg/day), with fifth lactation as the reference.

Lactation order	*n*	*β*	*SE*	*DF*	*t value*	***P***	*Lower 95% CI*	*Upper 95% CI*
1st lactation	455	-4.6	0.49	5851	-9.37	<0.001	-5.53	-3.62
2nd lactation	312	-1.1	0.49	5891	-2.15	0.03	-2.01	-0.09
3rd lactation	171	-0.4	0.51	5928	-0.76	0.45	-1.39	0.62
4th lactation	108	0.1	0.51	5929	0.21	0.84	-0.89	1.10
5th lactation*	84	0.0	0.00	-	-	-	-	-

Note: The column 'n (cows)' represents the number of individual cows in each lactation group included in the analysis. Positive β indicates higher ECM relative to the reference; negative β indicates lower ECM. β = estimate; SE = standard error; DF = degrees of freedom; CI = confidence interval. Statistically significant effects (*P*<0.05) are in bold. *The reference category (5th+) includes 56 cows in their 5th lactation and 28 cows in their 6th lactation.

**Table 3:** Estimated fixed effects for lactation phase on energy-corrected milk yield (ECM, kg/day), with 400 days in milk as the reference.

Covariate	*β*	*SE*	*DF*	*t value*	*P*	*Lower 95% CI*	*Upper 95% CI*
Days in milk (DIM)	-0.04	0.00	5597	-57.58	<0.001	-0.049	-0.05

Note: β = estimate; SE = standard error; *P* = *P*-value; DF = degrees of freedom; CI = confidence interval. Significant effects (*P*<0.001) are in bold.

**Table 4:** Estimated fixed effects for additional covariates on energy-corrected milk yield (ECM, kg/day).

Covariates	*β*	*SE*	*DF*	*t value*	*P*	*Lower 95% CI*	*Upper 95% CI*
LogSCC	-0.4	0.22	5945	-1.65	0.0990	-0.78	0.07
Urea	0.1	0.01	5877	10.00	<0.001	0.08	0.12

Note: Positive β indicates higher ECM relative to the reference; negative β indicates lower ECM. LogSCC = log-transformed somatic cell count; β = estimate; SE = standard error; DF = degrees of freedom; CI = confidence interval. Statistically significant effects (*P*<0.05) are in bold.

Interpretation of Intercept: The Intercept (β=40.4 kg/day) represents the baseline ECM yield for the reference group-specifically, a healthy cow in her 5^th^ lactation at 400 days in milk. All other β estimates in the table represent the daily milk loss or gain relative to this baseline.

The mixed-effects model revealed phase-specific effects on ECM for several lesion types. During treatment, ECM was significantly lower than in Healthy cows for digital dermatitis (β=−0.7 kg/day, t(4211)=−2.26, *P*=0.02), interdigital phlegmon (β=−4.8 kg/day, t(5375)=−2.47, *P*=0.01), white line disease (β=−1.6 kg/day, *P*=0.01), sole ulcer (β=−2.4 kg/day, *P*<0.001), toe ulcer (β=−2.3 kg/day, *P*<0.001), and combination lesions (β=−1.6 kg/day, *P*=0.03).

Digital dermatitis showed higher ECM post-treatment (β=+1.3 kg/day, t(3816)=3.91, *P*<0.001). White line disease had significantly higher ECM pre-treatment (β=+2.4 kg/day, *P*=0.01). This finding reflects the higher baseline production and associated increased susceptibility of these individuals to claw lesions (reverse causality), rather than suggesting a beneficial effect of the pre-clinical disease phase. Following treatment, white line disease showed a marginal post-treatment reduction (β=−1.4 kg/day, *P*=0.05). Interdigital dermatitis showed no significant phase effects (*P*>0.05).

During treatment, the largest ECM deficit relative to Healthy occurred in interdigital phlegmon (−4.8 kg/day; ≈−14.2%), followed by sole ulcer (−2.4; ≈−7.1%), toe ulcer (−2.3; ≈−6.8%), and white line disease/combination (each −1.6; ≈−4.7%), while digital dermatitis showed a smaller reduction (−0.7; ≈−2.1%); post-treatment, digital dermatitis exceeded Healthy (+1.3; ≈+3.8%) whereas sole ulcer remained below (−1.9; ≈−5.6%), and pre-treatment white line disease was above Healthy (+2.4; ≈+7.1%).

ECM differed by lactation order relative to the fifth lactation (reference). First-lactation cows produced substantially less ECM (β=−4.6 kg/day, t(5851)=−9.37, *P*<0.001), and a smaller but significant reduction was observed in the second lactation (β=−1.1 kg/day, t(5891)=−2.15, *P*=0.03). Differences for the third and fourth lactations were not statistically significant (*P*>0.05). Overall, ECM increased with parity and plateaued by later lactations, with the fifth lactation representing the highest reference level in this dataset.

Expressed relative to the Healthy LS mean (33.91 kg/day), ECM was lower by approximately −13.6% in first lactation and −3.2% in second lactation, with no meaningful percentage differences for third and fourth lactations.

Lactation phase, measured as days in milk (DIM), had a highly significant effect on ECM.

Specifically, ECM decreased with increasing DIM (β=–0.04 kg/day per day, t(5898)=–57.58, *P*<0.001), indicating a progressive decline in milk yield relative to the reference point of 400 days in milk.

Among the covariates, urea showed a positive association with ECM (β=0.10 kg/day per unit, *P*<0.001), whereas log-transformed somatic cell count (LogSCC) was negatively associated with ECM but did not reach statistical significance (β=−0.4 kg/day, *P*=0.099). Although LogSCC was non-significant in the final adjusted model, this trend aligns with the expected biological impact of subclinical inflammation on yield. The lack of statistical significance likely arises from the dilution effect in high-yielding cows, where larger milk volumes can mask inflammatory signals, an observation consistent with Guzzo et al. (2018). Expressed relative to the Healthy LS mean (33.91 kg/day), the urea coefficient corresponds to an increase of ≈+0.30% ECM per one-unit increase (*P*<0.001), whereas the LogSCC coefficient corresponds to ≈−1.18% per one log-unit (*P*=0.099, not significant). Any phase-related movement of SCC should therefore be interpreted cautiously within the adjusted model.

**Fig. 1:**
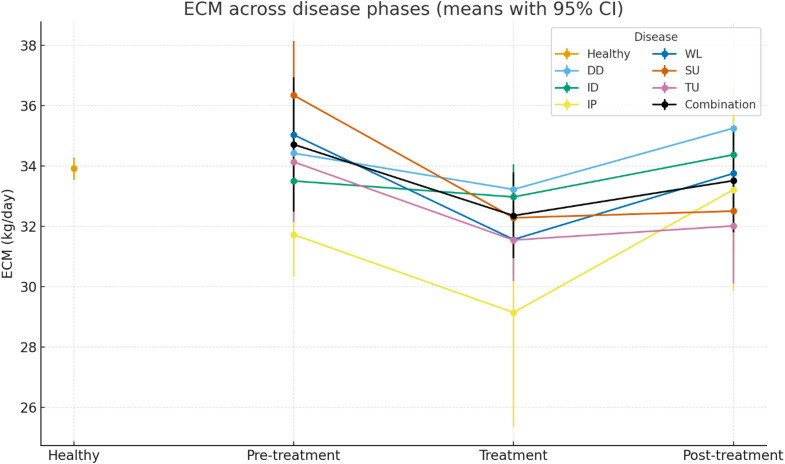
Mean energy-corrected milk (ECM, kg/day) across disease phases with 95% confidence intervals. For most diseases, ECM declines during treatment and partially rebounds post-treatment. Healthy cows are shown for reference (single point). Error bars denote 95% CIs.

Table 5 and Fig. 1 present least-squares means (LS means ± SE) of ECM by diagnosis and disease phase. Healthy cows maintained a stable ECM (33.91±0.19 kg/day). The lowest ECM level occurred in interdigital phlegmon (IP) during treatment (29.14±1.93 kg/day). Across all disease groups, ECM typically decreased during the treatment phase with partial recovery post-treatment. Digital dermatitis (DD) showed a decline during treatment (33.22±0.31 kg/day) followed by an increase post-treatment (35.25±0.34 kg/day). Similar treatment-phase depressions with partial post-treatment recovery were observed for interdigital dermatitis (ID), IP, white line disease (WL), sole ulcer (SU), toe ulcer (TU), and combination lesions. Overall, the pattern indicates a transient treatment-phase depression with diagnosis-specific recovery.

**Table 5:** Least-squares means (± SE) of energy-corrected milk (ECM, kg/day) by diagnosis and disease phase.

Diagnosis	Phase	ECM (kg/day)	95% CI
Healthy	Healthy	33.91 ± 0.19	33.54 - 34.28
Digital dermatitis	Pre-treatment	34.42 ± 0.39	33.65 - 35.20
	Treatment	33.22 ± 0.31	32.61 - 33.82
	Post-treatment	35.25 ± 0.34	34.58 - 35.92
Interdigital dermatitis	Pre-treatment	33.50 ± 0.77	31.99 - 35.02
	Treatment	32.97 ± 0.55	31.89 - 34.05
	Post-treatment	34.37 ± 0.62	33.15 - 35.59
Interdigital phlegmon	Pre-treatment	31.72 ± 0.71	30.33 - 33.11
	Treatment	29.14 ± 1.93	25.37 - 32.91
	Post-treatment	33.21 ± 1.70	29.87 - 36.56
White line disease	Pre-treatment	35.03 ± 0.83	33.40 - 36.66
	Treatment	31.56 ± 0.57	30.44 - 32.68
	Post-treatment	33.75 ± 0.66	32.46 - 35.03
Sole ulcer	Pre-treatment	36.34 ± 0.92	34.54 - 38.14
	Treatment	32.28 ± 0.63	31.04 - 33.51
	Post-treatment	32.50 ± 0.71	31.11 - 33.89
Toe ulcer	Pre-treatment	34.13 ± 1.01	32.14 - 36.12
	Treatment	31.54 ± 0.69	30.18 - 32.90
	Post-treatment	32.01 ± 0.97	30.10 - 33.92
Combination	Pre-treatment	34.71 ± 1.14	32.48 - 36.94
	Treatment	32.35 ± 0.73	30.93 - 33.78
	Post-treatment	33.51 ± 0.88	31.79 - 35.24

Note: Values are LS means ± SE from the mixed model; CI = confidence interval; ECM = energy-corrected milk.

From pre-treatment to treatment, the largest relative decline occurred in SU (−4.06 kg; −11.17%), whereas the strongest treatment-to-post rebound was seen in IP (+4.07 kg; +13.97%). Overall, IP ended slightly above its pre-treatment baseline (+1.49 kg; +4.70% pre→post), while SU remained the most depressed (−3.84 kg; −10.57% pre→post).

The overall Least-Squares Mean (LS Mean) for healthy cows (33.91±0.19 kg/day) represents the estimated average production when all continuous covariates (DIM, LogSCC, Urea) are set to their sample means (or a relevant representative value) and discrete covariates (Lactation Order) are averaged. This differs from the Intercept of 40.4 kg/day, which is anchored to the reference level of all fixed effects.

## Discussion

A key strength of this study, distinguishing it from several large-scale investigations (Amory et al., 2008; Cha et al., 2010; Green et al., 2010; Charfeddine and Pérez-Cabal, 2017), is the use of high-quality data from a single, high-performing dairy farm. While multi-farm studies offer broader generalizability, our single-herd design provides superior internal validity by eliminating confounding noise from varying management practices, nutrition, and environmental conditions. This consistency allows for a more precise estimation of how specific claw lesions directly impact ECM yield within a stable production environment.

This study shows a significant treatment-phase depression in dairy cow performance across specific claw lesions, with variable persistence post-treatment that is most evident for claw-horn lesions. Unlike general reports, we quantify production losses by diagnosis and disease phase, using each cow’s individual pre-disease baseline for context. However, the interpretation of this baseline requires caution. Green et al. (2002, 2010) demonstrated that milk yield in cows that eventually develop clinical lameness can begin to decline as early as four months before a formal diagnosis or treatment occurs. In our study, the 14-day pre-treatment window used as an individual baseline might therefore already reflect early subclinical production losses. This methodological challenge is consistent with the findings of Bicalho et al. (2008), who noted that cows often exhibit declining production trends several months before lameness is clinically detected. Their study demonstrated that when the baseline period is too close to the event, or if it does not account for the cow’s potential yield (e.g., using early lactation yield as a covariate), the true magnitude of the loss is likely underestimated (Bicalho et al., 2008).

This is particularly relevant for chronic conditions such as sole ulcers (SU), where the pathological process in the corium precedes clinical lameness by weeks. Consequently, the production drops we report (e.g., β ≈ -2.4 kg/day for SU) likely represent only the acute phase of a more prolonged depletion, potentially leading to an underestimation of the total cumulative loss throughout the entire disease process.

The largest treatment-phase decrease in ECM occurred in interdigital phlegmon (IP) (β ≈ −4.8 kg/day vs Healthy; Table 1), exceeding several earlier estimates of 1.5–4 kg/day (Warnick et al., 2001; Cha et al., 2010). Our findings contrast with those of Häggman et al. (2015), who did not observe a significant decrease in milk production during IP outbreaks in Finnish loose-housed herds, attributing the economic impact primarily to discarded milk following antibiotic treatment and involuntary culling. The pronounced production drop in our study likely reflects the high-yielding nature of the herd and a potentially more acute systemic response to the infection. As Häggman et al. (2015) emphasize, IP is a painful, necrotizing inflammation; in elite producers, the physiological redirection of nutrients toward immune defense and tissue repair can result in immediate and severe yield losses, even if clinical recovery is rapid after successful antimicrobial therapy.

Furthermore, our results show a more dramatic impact compared to Charfeddine and Pérez-Cabal (2017), who reported daily energy-corrected milk losses ranging from 1.47 to 2.66 kg for severe cases of sole ulcers and white line disease. This discrepancy is further highlighted when considering the daily losses reported by Robcis et al. (2023a) and the bioeconomic model of Robcis et al. (2023b), which estimated the average annual cost of a lame cow at €307.50, driven largely by a mean lameness duration of 10.3 weeks. While Robcis et al. (2023b) emphasize the long-term economic drain of chronic cases, the acute daily losses in our high-yielding environment are substantially higher. This suggests that in elite herds, the immediate collapse of the metabolic equilibrium leads to sharper production troughs than those estimated by broad-scale models or population studies. The magnitude of these losses likely reflects the high-yielding context of our herd (Healthy LS mean ≈ 33.91 kg/day).

Additional metabolic or inflammatory stress can cause sharper and more prolonged depressions, consistent with other high-yielding herds (Bruijnis et al., 2010; Solano et al., 2015) and recent evidence associated with sustained inflammation and impaired locomotion (Sadiq et al., 2024; Griffiths et al., 2024). This aligns with the position that production diseases in high-yielding cows are often a consequence of metabolic imbalance rather than high yield per se, where the redirection of energy towards immune competence and maintenance occurs at the expense of milk synthesis (Ingvartsen et al., 2003). Specifically, subclinical metabolic disorders such as ketosis and acidosis have been shown to cause significant immediate and carry-over losses in daily milk yield, with daily drops reaching up to 4.2 kg for ketosis and 2.8 kg for acidosis in high-producing Holstein cows (Gantner et al., 2016). Furthermore, the shape of the lactation curve is fundamentally altered by these metabolic challenges; transition period diseases not only reduce peak yield but also accelerate the rate of post-peak decline, leading to substantial cumulative losses throughout the entire lactation (Hostens et al., 2012). The systemic nature of these impacts is further evidenced by the link between rumen acidosis and the development of laminitis, where nutritional stress translates into structural claw damage and long-term production deficits (Teixeira Passos et al., 2023).

Whereas prior work often treated lameness as a generalised condition (Bicalho et al., 2009), our results demonstrate diagnosis-specific impacts: claw-horn lesions—sole ulcer (SU) and toe ulcer (TU)—showed substantial treatment-phase losses (≈ −2.4 and −2.3 kg/day, respectively; Table 1), while digital dermatitis (DD) produced a smaller but statistically significant reduction (≈−0.7 kg/day during treatment; Table 1). This hierarchy of impact—where non-infectious claw-horn lesions cause more severe production drops than infectious lesions like DD—is consistent with the findings of Charfeddine and Pérez-Cabal (2017), who reported energy-corrected milk losses of up to 2.66 kg/day for severe sole ulcers compared to much lower losses for dermatitis. Similarly, Amory et al. (2008) observed that sole ulcers were associated with the highest mean milk loss among all studied lesions. Our findings also align with Green et al. (2010), who noted that while most clinical lameness cases negatively affect yield, the magnitude is significantly higher for horn lesions due to their chronic nature and associated pain. Interestingly, Machado et al. (2010) demonstrated that cows with claw-horn disruption lesions in previous lactations are not only at higher risk of recurrence but also exhibit lower survivability and reduced milk production throughout the subsequent lactation, reinforcing the long-term systemic drain of these specific conditions. While Robcis et al. (2023a) reported lower average daily losses in their meta-analysis, they emphasized that in high-yielding environments—similar to our study herd—the acute phase of horn lesions triggers a sharper metabolic decline, explaining the more pronounced troughs we observed.

Using the pre-treatment phase as an individual baseline indicates that production begins to decline before clinical treatment starts. For TU, the difference between pre-treatment and post-treatment LS-mean ECM fell from 34.13±1.01 to 32.01±0.97 kg/day post-treatment (Table 5). A borderline persistent post-treatment deficit was evident for SU (β=−1.9; *P*=0.05; Table 1). The observation that production starts to falter even before formal intervention is critically supported by Green et al. (2002), who demonstrated that milk yield in cows subsequently diagnosed with lameness begins to decrease substantially before the clinical event. Specifically, their study found that the yield of "soon-to-be-lame" cows was significantly lower than that of healthy counterparts for at least several weeks prior to diagnosis, with the gap widening as the clinical event approached (Green et al., 2002). This reinforces the concern that our 14-day pre-treatment window, while providing an individual reference, may already be part of a downward trajectory.

Consequently, the production levels we used as a "baseline" might already reflect subclinical losses, leading to an underestimation of the true cumulative impact of these lesions. As Green et al. (2002) argue, clinical lameness is often the "tip of the iceberg" of a much longer period of impaired performance. This pattern, also supported by later findings (Green et al., 2010), suggests that the physiological stress and pain associated with developing lesions—particularly chronic ones like sole ulcers—initiate a metabolic redirection long before the cow is prioritized for treatment. This long-term impact on the lactation curve, particularly for sole ulcers, was also modeled by Cha et al. (2010), who emphasized that the economic cost of chronic lesions is driven more by this persistent yield depression than by the immediate costs of treatment. Together, these patterns support the view that pain and stress-related reductions in feed intake initiate the decline and can sustain negative impacts beyond active therapy; early detection is therefore critical because subclinical losses can accumulate before overt lameness is recognised (Schlageter-Tello et al., 2014).

A pre-treatment elevation relative to Healthy was observed for white line disease (WL) (β=+2.4; *P*=0.01; Table 1). This is consistent with Amory et al. (2008) and Green et al. (2002, 2010), who reported that 'ever-lame' cows, particularly those with lesions such as white line disease, were often significantly higher-yielding animals during their healthy periods. This reinforces the hypothesis that high-yielding animals are at greater innate risk of claw lesions due to higher metabolic demand and increased standing times required for feed intake.

From the LS-mean trajectories (Fig. 1; Table 5), the timing of treatment initiation likely varied. In some cases, treatment appears to have been initiated late, implying a longer subclinical period that could amplify treatment-phase losses and slow recovery. Acute, systemic conditions such as IP plausibly drive short-term catabolism and anorexia, producing a pronounced treatment-phase trough with variable rebound. In contrast, DD often responds rapidly to topical therapy and analgesia, enabling faster normalisation post-treatment. Claw-horn lesions (WL, SU, TU) showed treatment-phase depressions with incomplete normalisation in parts of the post-treatment period—consistent with residual pain and the time required for horn regrowth. This lack of full recovery is further explained by Machado et al. (2010), who found that claw-horn disruption lesions (CHDL) have a long-term debilitating effect, leading to significantly lower milk production and decreased survivability throughout the remainder of the lactation. The structural damage to the corium often results in permanent changes that hinder a return to peak performance. Furthermore, Griffiths et al. (2020) demonstrated that cows with chronic lesions often suffer from prolonged hyperalgesia and altered behavioral patterns, such as reduced feed intake, which persist long after the clinical lesion has been treated. Our observation that horn lesions (SU, WL) result in more severe and less reversible losses compared to infectious lesions like DD aligns with the hierarchy of impact reported by Charfeddine and Pérez-Cabal (2017) and Amory et al. (2008).

Several mechanisms likely constrain full restitution to pre-disease output. As demonstrated by Newsome et al. (2016), chronic lameness and CHDL are associated with the development of new bone growth (exostosis) on the caudal aspect of the distal phalanx. These permanent bony changes can cause ongoing trauma to the sensitive corium from within, even after the initial external lesion has healed, creating a cycle of inflammation and structural vulnerability (Newsome et al., 2016). This is further compounded by the findings of Bicalho et al. (2009), who highlighted the critical role of the digital cushion. Their study showed that a reduction in the thickness of the digital cushion, often linked to low body condition score or previous lameness episodes, significantly increases the risk of CHDL. Once this fat pad is compromised or the suspensory apparatus is stretched, the cushioning capacity of the claw is permanently reduced, leading to persistent discomfort and a failure to return to pre-disease production levels (Bicalho et al., 2009).

The disruption of time budgets is a critical factor; as Gomez and Cook (2010) demonstrated, lame cows significantly alter their daily behavioral patterns, often increasing standing time in alleys and reducing lying time, particularly in restrictive environments. This altered behavior, coupled with the gait changes and abnormal loading described by Cook et al. (2004), perpetuates microtrauma to the sensitive laminae and digital cushion, further hindering the healing process.

Additionally, these impacts are strongly moderated by parity and stage of lactation (DIM). High-producing cows in early lactation are particularly vulnerable due to the physiological demands of peak yield and the concurrent softening of the suspensory apparatus around calving. Collard et al. (2000) found that cows with higher genetic merit for milk production may be at an inherently greater risk of health disorders, including lameness, because they prioritize nutrient partitioning toward the udder. This prioritization, often occurring during periods of negative energy balance, can compromise the structural integrity of the claw, reinforcing the systemic nature of the disease.

Together, these factors—along with slow horn regrowth, residual structural damage to horn/corium, persistent nociception that depresses intake and alters time budgets, and altered gait and loading that perpetuate microtrauma—plausibly maintain modest but persistent ECM deficits even after clinical resolution (Randall et al., 2015), with previous estimates suggesting that production may remain compromised for up to five months post-treatment (Green et al., 2002). More generally, lameness should be viewed as a systemic challenge rather than a purely local lesion. Pain and inflammation activate the hypothalamic–pituitary–adrenal axis, elevating cortisol and redirecting energy and nutrients away from lactation toward immune defence and tissue repair—responses also documented for other systemic diseases such as mastitis (Džermeikaitė et al., 2025; Ingvartsen and Moyes, 2013).

In our high-yielding herd, where the metabolic balance between intake and output is precarious, such physiological prioritisation is sufficient to depress milk output even when overt clinical signs are only emerging. Additional metabolic or inflammatory stress can therefore cause sharper and more prolonged depressions, consistent with other high-yielding herds (Bruijnis et al., 2010; Solano et al., 2015).

The adjusted LogSCC effect in our mixed model was negative and non-significant (β=−0.4; *P*=0.099; Table 4), indicating a minimal isolated association after accounting for diagnosis and DIM. Apparent paradoxes in SCC–yield relationships can arise from dilution in high-yield settings—larger volumes can mask early inflammatory signals (Guzzo et al., 2018). However, the relationship between udder health and claw integrity likely extends beyond simple cell counts. As suggested by recent evidence, the severity and etiology of mastitis may play a more decisive role in claw health than SCC alone. Faustmann et al. (2025) demonstrated a significant association between acute Escherichia coli mastitis and the development of acute laminitis. The systemic release of endotoxins (lipopolysaccharides) during severe gram-negative mastitis can trigger a cascade of inflammatory mediators that disrupt the microcirculation within the claw corium, leading to structural failure. This systemic link is further supported by Griffiths et al. (2020), who highlighted that systemic inflammatory events, including severe mastitis, can lead to the degradation of the suspensory apparatus of the distal phalanx. This degradation facilitates the sinking and tilting of the pedal bone, which is a primary step in the pathogenesis of claw-horn disruption lesions (CHDL). Therefore, a high-severity mastitis score (e.g., score 3) or the presence of coliform bacteria likely exerts a far greater systemic insult on the claw laminae than chronic, low-grade subclinical mastitis reflected by moderately elevated SCC.

Any phase-related co-movement of SCC should therefore be interpreted cautiously in the context of the adjusted model (Table 4). Our estimates represent differences relative to the Healthy reference within phase (Table 1) and LS-means anchored to each cow’s pre-disease baseline (Table 5). While this aids interpretation, residual confounding cannot be excluded. Unmeasured factors such as body condition score (BCS), energy balance, and heat load likely moderate the observed effects. For instance, Machado et al. (2010) and Bicalho et al. (2009) established a clear link between low BCS and a reduction in the thickness of the digital cushion, which significantly increases the risk of CHDL. This is further compounded by the negative energy balance typical of early lactation, which, as demonstrated by Collard et al. (2000), compromises overall health and redirects nutrients away from structural maintenance toward milk synthesis. Furthermore, environmental factors like heat load can exacerbate these impacts; Cook et al. (2004) and Gomez and Cook (2010) observed that heat-stressed cows alter their time budgets by standing longer in alleys to facilitate cooling, thereby increasing the mechanical load on the claws and predisposing them to laminitis-related injuries. Therefore, while our model accounts for diagnosis and DIM, the interplay of these physiological and environmental stressors—as documented in the aforementioned studies—merits cautious interpretation of yield depressions, especially where borderline *P*-values are concerned. Aggregating days 22–120 into one post-treatment phase may average cows at different recovery stages, attenuating observed rebounds.

A primary limitation of this study is that data were derived from a single high-performing dairy farm. While this design ensured high internal validity by eliminating confounding noise from varying nutrition, genetics, and environmental conditions, it may limit the external generalizability (external validity) of the absolute loss magnitudes. Differences in housing systems (e.g., deep-bedded packs vs. concrete alleys), flooring types, claw-trimming protocols, and regional management practices could influence both lesion prevalence and the dynamics of recovery. For instance, cows on rubber mats or pasture might exhibit different recovery trajectories compared to those on solid concrete. Therefore, while the diagnosis-specific hierarchy of production losses identified here provides a robust framework for understanding claw lesions impact, caution should be exercised when extrapolating these specific effect sizes to dairy systems with substantially different environmental or management profiles.

## Practical implications and future perspectives

Economic incentives are central to adoption of preventive measures (Dutton-Regester et al., 2019). As demonstrated in the comprehensive meta-analysis by Robcis et al. (2023a), the economic burden of lameness is not only substantial but also highly variable depending on the lesion type and production system. Furthermore, bioeconomic modeling by Robcis et al. (2023b) suggests that the true cost of a clinical case—often exceeding €300—is driven primarily by long-term milk loss and increased culling risk, rather than just treatment costs. Effective prevention, therefore, requires a shift toward proactive management. Saro et al. (2024) emphasize that integrated preventive strategies, including automated monitoring and optimized environment design, offer a high return on investment by preserving the cow's peak performance and longevity.

Our findings reinforce this value, suggesting that earlier detection, prompt lesion-specific treatment, and effective analgesia are not merely welfare necessities but critical economic drivers in high-performing herds. The importance of rapid intervention is underscored by Leach et al. (2012), who showed that early treatment significantly reduces the risk of mild lesions progressing to chronic clinical lameness. Furthermore, a randomized controlled trial by Thomas et al. (2015) demonstrated that the most effective recovery protocol for claw-horn disruption lesions (CHDL) involves a combination of therapeutic trimming, the application of a claw block, and a course of non-steroidal anti-inflammatory drugs (NSAIDs). The inclusion of analgesia is vital not only for welfare but also for performance; Gundelach et al. (2013) found that NSAID administration supports the recovery of milk yield by reducing the systemic impact of pain-related stress.

Beyond acute therapy, sustained support through convalescence is equally important. As Thomsen et al. (2023) highlighted in their systematic review, factors such as stall comfort, flooring traction, and dedicated claw-care follow-up are decisive for long-term recovery and the prevention of recurrence.Severe lameness can cost hundreds of euros per case through lost milk, culling, and veterinary costs (Bruijnis et al., 2010), underlining the return on prevention, especially for persistent claw-horn lesions.

Preventive programmes—regular claw trimming, flooring design, optimised nutrition, and genetic selection for claw-health traits—offer meaningful potential to reduce losses (Ring et al., 2018). Future work should assess the integration of precision technologies for early detection, the role of nutritional strategies in supporting claw-horn integrity, and the long-term economic returns of preventive interventions. Genetic selection remains a promising avenue, with evidence for heritable variation in claw-lesion susceptibility and locomotion scores (van der Spek et al., 2013). Recent genomic studies further support this by identifying specific markers for claw-horn lesion resistance, suggesting that single-step genome-wide association analyses can significantly accelerate genetic progress in herd mobility (Li et al., 2023).

## Conclusion

This study confirms that claw lesions causes clear, diagnosis – and phase-specific depressions in milk production, with recovery often incomplete—particularly for claw-horn lesions. This pattern is consistent with a systemic physiological burden, not just a local problem, with effects persisting significantly beyond clinical treatment.

To mitigate these economic and welfare losses, key priorities for practical management include:

• Early and Targeted Therapy: Prioritizing the identification of subclinical cases and timely, lesion-specific treatment combined with effective analgesia.
• Robust Preventive Measures: Implementing regular functional trimming, optimizing flooring, traction, cow flow, and nutrition to support claw integrity.
• Long-term Genetic Strategy: Incorporating genetic selection for improved claw-health traits into breeding programs.

Future research should further investigate precision methods for earlier identification of subclinical cases, quantify the impact of time-to-treatment and time-to-recovery, and evaluate the long-term cost–benefit of preventive interventions in high-producing herds. Collectively, addressing these points is crucial for protecting milk yield, economic stability, and animal welfare.

## Ethical statement

**Project Title:** Impact of Claw Disease on Dairy Cow Energy-Corrected Milk Yield: Differential and Persistent Effects up to 120 Days Post-Treatment

The authors confirm that the ethical policies of the journal, as noted on the journal’s author guidelines page, have been adhered to.

**Ethical approval:** This study was conducted in accordance with the European Union Directive 2010/63/EU and national legislation for the protection of animals used for scientific purposes. Since the study was retrospective and based on existing production and health records routinely collected during standard farming and veterinary practices, and no animals were subjected to any experimental procedures or interventions beyond routine clinical care, formal ethical approval from an institutional ethics committee was not required.

**Animal Welfare:** All hoof treatments and diagnostic procedures were performed by qualified farm personnel and veterinarians as part of standard herd health management. No animals were harmed or specifically manipulated for the purpose of this research.

## CRediT authorship contribution statement

**Zdeněk Havlíček:** Writing – review & editing, Supervision, Resources, Funding acquisition, Conceptualization. **Lucie Langová:** Writing – original draft, Visualization, Project administration, Investigation, Formal analysis. **Irena Vrtková:** Validation, Methodology, Data curation. **Petr Doležal:** Writing – review & editing, Validation, Resources. **Petr Kouřil:** Software, Investigation, Data curation. **Katarzyna Szwedziak:** Writing – review & editing, Resources.

## Declaration of competing interest

The authors declare that they have no known competing financial interests or personal relationships that could have appeared to influence the work reported in this paper.

The research was conducted in the absence of any commercial or financial relationships that could be construed as a potential conflict of interest.
